# Sub-Saharan African Prostate Cancer Patient-Derived Cell Lines and High-Throughput Drug Screening: Addressing Ancestry Underrepresentation in Oncobiology Research

**DOI:** 10.3390/cancers18152452

**Published:** 2026-07-30

**Authors:** Carla S. Dos Santos, Ana C. Magalhães, Veronica Fernandes, António Pombinho, Lurdes Torres, Margarida André, Adelaide Sousa, Pedro Sequeira, Daniel Pinto, Cláudia Pereira, Paulo M. Costa, Lúcio Lara Santos, Luisa Pereira

**Affiliations:** 1 i3S—Instituto de Investigação e Inovação em Saúde, Universidade do Porto, 4200-135 Porto, Portugal; carlas@i3s.up.pt (C.S.D.S.); acmagalhaes@i3s.up.pt (A.C.M.); vfernandes@i3s.up.pt (V.F.); antonio.pombinho@i3s.up.pt (A.P.); 2 IPATIMUP—Instituto de Patologia e Imunologia Molecular da Universidade do Porto, 4200-135 Porto, Portugal; 3 ICBAS—Instituto de Ciências Biomédicas Abel Salazar, Universidade do Porto, 4050-313 Porto, Portugal; 4 Instituto Português de Oncologia do Porto, 4200-072 Porto, Portugal; mlurdes.torres@ipoporto.min-saude.pt (L.T.); llarasantos@gmail.com (L.L.S.); 5 Unidade Local de Saúde Almada-Seixal, 2805-267 Almada, Portugal; margarida.andre@ulsas.min-saude.pt (M.A.); adelaide.sousa@ulsas.min-saude.pt (A.S.); pedro.ms.sequeira@gmail.com (P.S.); daniel.pinto@hgo.min-saude.pt (D.P.); claudia.pereira@ulsas.min-saude.pt (C.P.); paulomatoscosta@gmail.com (P.M.C.); 6 NOVA Medical School, NOVA University of Lisbon, 1169-056 Lisboa, Portugal; 7 Faculdade de Medicina, Universidade de Lisboa, 1649-028 Lisboa, Portugal; 8 ONCOCIR—Education and Care in Oncology, PALOP—Lusophone Africa, 4200-072 Porto, Portugal

**Keywords:** prostate cancer, patient-derived cell lines, sub-Saharan African ancestry, high-throughput drug screening

## Abstract

**Background/Objectives**: Prostate cancer (PC) exhibits marked disparities in incidence and mortality across ethnicities, with men of Sub-Saharan African (SSA) ancestry experiencing 1.7 and 2.0 times higher values, respectively, than European men (EUR). However, SSA preclinical models remain scarce (just one commercial cell line). In this study, we established and characterized a novel panel of PC cell lines derived from SSA patients using conditional reprogramming (CR), a method that enables efficient propagation of primary cells while maintaining their genotypic and phenotypic features. **Methods**: CR was applied to five SSA-PC samples, and successfully propagated samples were authenticated by STR and ~1 million SNP profiling, and extensively characterized for proliferative capacity, migratory behaviour, karyotyping and epithelial and prostate tumour lineage markers. To explore drug response profiles, a high-throughput screen (HTS) of 1280 clinically annotated compounds was conducted. **Results**: Three SSA-PC cell lines were successfully established and authenticated, and five potential drug hits were validated. A new finding was the reduced sensitivity of SSA-derived models (9.0 times difference compared to commercial EUR PC cell lines) to camptothecin, a TOP1 inhibitor, while being equally sensitive to epirubicin hydrochloride, a TOP2 inhibitor. The cardiac glycoside digoxin, anthelmintic pyrvinium pamoate and antirheumatic agent auranofin were also efficient drugs in the in vitro testing. **Conclusions**: These results support the relevance of SSA-derived PC models for preclinical drug screening and highlight the value of including ancestry-diverse models in oncobiology research.

## Simple Summary

Prostate cancer affects men of Sub-Saharan African ancestry at disproportionately higher rates and with worse clinical outcomes, yet only one commercial cell model from this ancestry exists to investigate this disparity. In this work, we established three new prostate cancer cell lines derived from Sub-Saharan African patients by conditional reprogramming. These cell lines were thoroughly characterized and used to screen over a thousand clinically annotated compounds in a high-throughput drug screening. Notably, the Sub-Saharan African-derived models showed reduced sensitivity (9.0 times) to camptothecin, a topoisomerase I inhibitor, compared to commercially available European prostate cancer cell lines, while sensitivity to epirubicin, a topoisomerase II inhibitor, was identical. These findings underscore the value of ancestry-diverse preclinical models in uncovering population-specific drug vulnerabilities and advancing more equitable cancer research.

## 1. Introduction

The research community has emphasized the urgent need to increase Sub-Saharan African (SSA) ancestry representation in omics studies, for both comprehensive diversity characterization and the study of complex diseases, as well as for the creation of informative in vitro models, including cancer cell lines, induced pluripotent stem cells (iPSCs) and organoids (reviewed in [1,2,3,4,5,6]). In fact, SSA samples (mainly from African Americans, AAs) account for less than 2.4% in genome-wide association studies (GWASs [7]), 12% in The Cancer Genome Atlas (TCGA [8]), 6% in cancer cell line panels [9], and 2% in iPSCs from the iPSCORE resource [10]. Efforts to counterbalance these biases have been conducted in the United States of America (USA; e.g., All of Us Research Program [11]) and, with more potential, across the African continent. In fact, it is increasingly obvious that AA samples are a poor proxy for the richness of diversity for human genomics and environmental factors (including the pathogenic landscape) across the African continent. The Human Heredity and Health in Africa (H3Africa) consortium have been paradigmatic in reducing the SSA data gap, by contributing high-throughput genomic data across the African continent (e.g., [12,13,14]) with many insights on SSA genomic susceptibility to complex diseases [15,16,17]. In the context of cancer, independent groups have been publishing omics studies for African cancer cohorts, especially for cancer types and subtypes that are more frequent and aggressive in the SSA versus European ancestry, such as triple-negative breast cancer [18,19,20] and prostate cancer (PC [21,22,23]).

While efforts to conduct omics characterization of SSA samples have advanced considerably in recent years, progress in closing the gap in the development of advanced in vitro models derived from SSA samples has been comparatively slower. A primary reason for this is the fact that extracted DNA can be safely sent intra- and inter-continently for high-throughput sequencing (after ethics and transfer permits are dealt with; e.g., [24]), while challenges with the transfer of viable cells within and outside Africa seem still insurmountable. Also, the field of in vitro modelling implies considerable dedicated lab resources and specialized expertise. So far, the literature reports some groups in South Africa who have the expertise to locally establish these advanced models from cancer patients [3,25,26]. Our group is advancing in collecting tumour samples from patients of nationalities from the Portuguese-speaking African Countries (PALOP), and propagating them in the lab [2]. In a first attempt, we are collecting these samples in Portuguese hospitals, from PALOP patients being treated locally, taking advantage of our close-by fully equipped laboratory conditions and expertise. This approach has the additional advantage of sampling individuals from six countries (Angola, Cape Verde, Guinea Bissau, Mozambique, Republic of Equatorial Guinea, and São Tomé and Príncipe), potentiating SSA continental diversity in our collection.

PC is an excellent case study to establish representative SSA in vitro models. SSA ancestry is a significant risk factor for PC, as shown in the controlled setting of the USA, where 2019 data for age-standardized incidence rates of PC per 100,000 men were 119.2 for European Americans (EAs) and 197.9 for AAs, while age-standardized mortality rates of PC per 100,000 men were 17.9 for EAs and 36.6 for AAs (cited in [23]). Despite these higher values among AA men, only one SSA cancer cell line is currently available at the Cancer Cell Line Encyclopedia [27], severely limiting functional studies and translational applications in PC for SSA ancestry. The establishment of SSA-representative PC in vitro models would provide a critical resource for interrogating the functional impact of the population-specific genetic determinants of PC in SSA men that are now being elucidated. Janivara et al. [23] conducted a genome-wide association study in 3963 PC cases and 3509 controls from Ghana, Nigeria, Senegal, South Africa and Uganda, identifying 15 statistically significant associations at 8q24.21, 6q22.1 and 11q13.3. These regions are known to contain cancer loci, such as variants at 8q24.21 that are close to long noncoding RNA genes that influence cell proliferation, metastasis and resistance to treatment (*PCAT1*, *PCAT2*, *PRNCR1*, *CASC19* and *CCAT1*), and that may modulate the expression of *c-MYC*. Significant hits at 6q22.1 are located in the intronic region of *RFX6* (correlated with tumour progression, metastasis and biochemical relapse of PC when upregulated by *HOXB13*) and in the exons of *GPRC6A* (accelerates PC proliferation). The 11q13.3 SNP hits are proximal to *MYEOV* (hominid-specific oncogene implicated in multiple cancers). Jaratlerdsiri et al. [21] conducted whole-genome sequencing (WGS) of treatment-naïve PC samples from 123 South African men, concluding with findings of African-ancestry-specific elevated tumour mutational burden, a greater number of predicted damaging mutations, a higher total of mutational signatures, driver genes [*NCOA2*, *WHR1*, *DDX11L1*, *PCAT1* (at 8q24.21, identified in the previous work) and *SETBP1* (close to 6q22.1)], and global mutational subtypes (GMSs) that feature copy-number gain (GMS-B) and are mutationally noisy (GMS-D). The same team [22] conducted the analyses of structural variants in the same WGS data, reporting that duplication events were 1.6- (relative frequency) to 2.5-fold (count) increased in African-derived tumours, being associated with *CDK12* inactivation and *MYC* copy-number gain, and deletion events associated with *SPOP* mutation; they also identified specific fusions between *TMPRSS2* and *LINC01525*, *FBXO7*, *GTF3C2*, *NTNG1* and *YPEL5*; and found 74 somatic SV hotspots impacting 18 new candidate driver genes, with *CADM2*, *LSAMP*, *PTPRD*, *PDE4D* and *PACRG* having therapeutic implications for African patients.

Whereas survival rates for PC have improved markedly in high-income countries (HICs), SSA countries continue to report high mortality. Globocan 2022 [28] estimated age-standardized mortality rates of 22.1 per 100,000 males in SSA, compared with 8.7 per 100,000 males in HICs. There is an urgent need to establish SSA patient-derived PC in vitro models that enable high-throughput screening (HTS) of drugs. Illustrating the potential of this approach, a limited drug screen of 30 compounds performed in South Africa using leukaemia patient-derived cells [26] revealed that irinotecan (a camptothecin analogue and topoisomerase I inhibitor typically used in solid tumours) was effective across many patient samples, outperforming conventional leukaemia therapies such as nilotinib. The study also identified synergistic effects in subsets of samples for combinations including nilotinib with irinotecan, fludarabine, or cladribine. In light of the limited representation of SSA origin among PC cell lines in the CCLE (1 in 10), conclusions derived from the pharmacological profiling of 24 anticancer drugs across the CCLE panel [27] warrant cautious interpretation when applied to SSA ancestry.

In this work, we established PC patient-derived cells from PALOP nationals, collected at biopsy or surgery, and both treatment-naïve. The cells were propagated in the lab through conditional reprogramming (CR [29]), a technique that combines culturing cells with growth-arrested (by irradiation) mouse fibroblast feeder cells in the presence of a Rho kinase (ROCK) inhibitor (Y-27632). The CR method offers numerous advantages, including preservation of both genotypic and phenotypic features of the samples, maintenance of tumour heterogeneity and reversibility of the process [30]. The successfully established PC cell lines were thoroughly characterized at both the molecular and phenotypic levels and subjected to HTS using the 1280-compound Prestwick Chemical Library.

## 2. Materials and Methods

### 2.1. Sample Collection and Tissue Preservation

PC tissue samples were collected at biopsy (transrectal ultrasound-guided) or surgery, without neoadjuvant therapy, from five patients at Unidade Local de Saúde Almada-Seixal, Portugal. These patients were selected for their SSA ancestry, and they gave informed consent to participate in this study. The study was approved by the Ethics Committee of Unidade Local de Saúde Almada-Seixal (Protocol 106/2021). For the biopsy, a high-frequency transrectal ultrasound probe was used to visualize the prostate in axial and sagittal planes, allowing assessment of gland volume and anatomical landmarks. Following antibiotic prophylaxis and local anaesthesia with a periprostatic nerve block, tissue cores were sampled using an automated spring-loaded biopsy needle. Transrectal ultrasound-guided biopsy was performed according to the established technique [31], using a systematic 12-core sampling scheme in line with current guideline recommendations [32]. Cores were obtained bilaterally from the base, mid-gland, and apex, with predominant sampling of the peripheral zone. For the purposes of this study, one additional biopsy core was obtained from each prostate region after completion of the standard systematic sampling to ensure the availability of tumour-containing tissue for research analyses; these additional cores did not alter the standard diagnostic workflow and did not compromise histopathological diagnosis. The collected tissue samples were washed with cold (4 °C) 1× PBS + 0.2% Primocin (InvivoGen, Toulouse, France) and inserted individually in a cryovial containing cryopreservation medium (90% FBS, 10% DMSO, 10 µM ROCK inhibitor and 100 µg/mL Primocin), and the cryovial was maintained in a slow-freezing container (Mr Frosty; Thermo Fisher Scientific, Waltham, MA, USA) at −80 °C for at least 24 h, and then kept outside the container, at the same temperature, until transport in dry ice.

### 2.2. Feeder Cell Culture, Irradiation and Conditioned Medium

Mouse fibroblasts 3T3-J2 (#P0011008, AddexBio, San Diego, CA, USA) were maintained in culture at 37 °C and 5% CO_2_ in DMEM (Thermo Fisher Scientific, Waltham, MA, USA) supplemented with 10% Foetal Bovine Serum (FBS—Biowest, Nuaillé, France) and 1% Penicillin–Streptomycin (Pen/Strep—Gibco, Waltham, MA, USA). When the feeder cells attained a 70–90% confluency, cells were detached, resuspended in F medium, and irradiated with 70 Gy, using a Gammacell 1000 SN 271 (Nordion, Ottawa, ON, Canada) with gamma rays, based on the protocol by Liu et al. [29,33]. The F medium consisted of 75% complete DMEM, 25% F12 (Gibco, Waltham, MA, USA), 0.125 ng/mL EGF (Thermo Fisher Scientific, Waltham, MA, USA), 25 ng/mL hydrocortisone (Sigma, Darmstadt, Germany), 0.25 µg/mL Amphotericin (Merck, Darmstadt, Germany), 5 µg/mL insulin (Merck, Darmstadt, Germany), and 8 ng/mL Cholera Toxin (Merck, Darmstadt, Germany), and was supplemented at the moment of use with 10 µM Y-27632 (ROCK inhibitor—Neo Biotech, Nanterre, France).

The irradiated feeder cells could be used directly in co-culture with the isolated primary epithelial cells, or to produce conditioned medium needed for when it is essential to have only the epithelial cells present (two passages in conditional medium before, e.g., karyotyping, genotyping, immunohistochemistry characterization and drug screening). In order to obtain the conditioned medium, the irradiated feeder cells were plated in T75 flasks, and maintained in culture for 72 h. At this timepoint, the medium was collected, centrifuged (448 RCF for 5 min), and filtered with a 0.22-µm PES filter (Thermo Fisher Scientific, Waltham, MA, USA). The final conditioned medium is made of three parts of this filtered medium mixed with one part of F medium and supplemented with 10 µM Y-27632 at the moment of use.

### 2.3. Isolation of Primary Cells and Propagation by Conditional Reprogramming

The tissues were propagated by conditional reprogramming, following mostly the protocol published by Liu et al. [29]. First, the epithelial cells were isolated, by mincing and digestion. After thawing at room temperature, the tissue samples were minced into small pieces and washed several times with cold (4 °C) 1× PBS + 0.2% Primocin till the solution was transparent. Then, digestion with 1 mg/mL Collagenase/Dispase (Roche, Basel, Switzerland) was conducted, under agitation at 37 °C, for around 1 h or until the solution was viscous without solid pieces. Then the solution stayed stagnant till two phases were visible, after which: (1) the supernatant was recovered, diluted in F medium, and centrifuged (448 RCF 5 min), with the resulting pellet being resuspended in F medium and then plated in one well in a 6-well plate containing irradiated feeder cells (1 × 10^5^ cells); (2) the sediment, still containing pieces of tissue, was digested again with TrypLE Express (Gibco, Waltham, MA, USA) for 20 min, with agitation at 37 °C, repeating the described process and resulting in a second pellet being plated in another well.

This co-culture, irradiated feeder cells and epithelial PC cells, remained in the incubator at 37 °C in 5% CO_2_, until confluent (10–15 days). Then, a differential trypsin treatment was conducted: (1) trypsin was added for 30 s–1 min, leading to detachment of the feeder cells that were discarded (visualized under a bright-field microscope); (2) the remaining epithelial cells were subjected to another trypsinization for 5 min at 37 °C, for their detachment. Around 1 × 10^6^ epithelial cells were plated in a T25 flask for a second passage of co-culture with irradiated feeder cells (2.5 × 10^5^ cells), and the process continued till growth stopped. The surplus epithelial cells in all the passages were resuspended in cell cryopreservation medium (90% FBS, 10% DMSO and 10 µM Y-27632) and cryopreserved at −80 °C (backup vials of lower passages were transferred to liquid nitrogen).

### 2.4. Culture of the Commercial PC Cell Lines

The commercial PC cell lines 22RV1 (ATCC, #CRL-2505; ATCC, Manassas, VA, USA) and PC-3 (ATCC, #CRL-1435) were propagated in RPMI-1640 medium (Gibco, Waltham, MA, USA) supplemented with 10% FBS and 1% Pen/Strep. Cells were maintained at 37 °C, under a 5% CO_2_ humidified atmosphere. The Cellosaurus database (https://www.cellosaurus.org/; consulted on 23 December 2025) indicates the following ancestry profiles for these commercial cell lines: 22RV1 with 98.08% European, 1.02% Asian, and 0.90% African; PC-3 with 97.87% European, 1.71% Asian and 0.41% African.

### 2.5. DNA Extraction, Genotyping (STR and DNA Microarray) and Check for Mycoplasma Contamination

DNA was collected in the initial passages for each cell line, from around 1 × 10^6^ cells. Cells were detached and centrifuged in order to form a pellet, to be used in the extraction by using the QIAamp DNA Mini Kit (Qiagen, Hilden, Germany) following the manufacturer’s instructions. The DNA concentration and purity were analysed first with a NanoDrop 1000 Spectrophotometer (Thermo Fisher Scientific, Waltham, MA, USA) and then with a Qubit™ 3 Fluorometer (Thermo Fisher Scientific, Waltham, MA, USA).

STR genotyping was done for the newly established cell lines and the commercial ones, with the kits PowerPlex^®^ 16 HS System (Promega, Madison, WI, USA) for sixteen loci (Penta E, D18S51, D21S11, TH01, D3S1358, FGA, TPOX, D8S1179, vWA, Amelogenin, Penta D, CSF1PO, D16S539, D7S820, D13S317 and D5S818) and the CLA IdentiFiler™ Plus PCR Amplification Kit (Applied Biosystems, Waltham, MA, USA) for sixteen loci (D18S51, D21S11, TH01, D3S1358, FGA, TPOX, D8S1179, vWA, Amelogenin, CSF1PO, D16S539, D7S820, D13S317, D5S818, D2S1338 and D19S433). The samples were run in a 3500xL Dx Genetic Analyzer (Thermo Fisher Scientific, Waltham, MA, USA). Minor alleles with less than 20% signal (but over 10%) relative to the major allele were marked with “+”, while peaks with a signal less than 10% of the major allele were not called. The STR profiles were compared against a database of STR profiles for commercial cell lines using the CLASTR 1.4.4 tool [34].

The newly established cell lines were screened for >850,000 variants included in the Axiom™ Precision Medicine Diversity Array (PMDA, Thermo Fisher Scientific, Waltham, MA, USA), which was genotyped in the GeneTitan MC Fast Scan Instrument (Thermo Fisher Scientific, Waltham, MA, USA). The software ADMIXTURE v1.3.0 [35] was applied to the profiles of the cell lines after merging with a panel of worldwide populations from the 1000 Genomes project (1kGP [36]) and pruning for LD by removing any SNP that had an r2 > 0.2 with another SNP, within a 50-SNP sliding window with steps of 5 SNPs. The ADMIXTURE was limited to a maximum likelihood for components (K) equal to 3, at the level of the main population groups, SSA, Eurasian and East Asian. Admixture results were visualized using the KronaPlots tool (v2.8.1. GitHub). RFMix v2 software [37], which infers ancestry for each segment of the genome between admixture of putative ancestral panels of haplotypes, was used in the unpruned and phased data set from the three SSA samples. The phased data from the 1kGP populations of Great Britain (representing Eurasian ancestry), Yoruba from Nigeria (SSA ancestry), and Han Chinese in Beijing (East Asian ancestry) were used as parental populations. The phasing of the SSA samples was obtained with SHAPEIT v2.r79044 [38] using the 1kGP phased data [36] as a reference panel and the HapMap phase 2 genetic map [39]. The admixture mapping visualization along the chromosomes was performed using the TAGORE tool (v1.1.2. GitHub). The mitochondrial DNA (mtDNA) haplogroups were inferred by using the HaploGrep tool (version 2 [40]), while for Y-chromosome haplogroups, the tool yhaplo was used [41]. These genomic profiles were also used to infer the polygenic risk scores (PRSs) for the three SSA-PC cell lines based on the associated variants described in Janivara et al. [23] (we did not include the non-binary variants, the variants located in the X chromosome, or rs879332972 not detected in 1kGP, ending up with 83 out of the 90 variants listed in their Supplementary Table S2). We used Michigan Imputation Server 2 (https://imputationserver.sph.umich.edu/#!, accessed on 21 July 2026) and the panel 1kGP Phase3 30× (GRCh38/hg38) as references for imputation, followed by PRS calculation in Plink 2.0 (https://www.cog-genomics.org/plink/2.0/, accessed on 21 July 2026) using the flag “—score”. The PRSs for the Yoruba males from 1kGP were also estimated to serve as an SSA population comparison.

All cells in culture were routinely screened for mycoplasma contamination, by PCR using the primer set MGSO/GPO1 and conditions from [42].

### 2.6. Karyotype

For karyotyping [43], cells were treated with colcemid (100 ng/mL; Gibco, Waltham, MA, USA) for 4 h at 37 °C to arrest cells in metaphase, then they were harvested and subjected to hypotonic treatment with 0.05 M KCl for 30 min at room temperature. Cells were fixed with freshly prepared methanol–acetic acid (3:1, *v*/*v*), washed and stored at −20 °C until slide preparation. Fixed cell suspensions were dropped onto chilled slides and air-dried overnight. Chromosome banding was performed by trypsin treatment followed by Leishman staining. Metaphases were automatically captured using the GSL-120 slide scanning system (CytoVision v7.4; Leica Biosystems, Nussloch, Germany), and karyotypes were analysed using CytoVision software. Multiple metaphases were evaluated per sample. Karyotypes were described according to the International System for Human Cytogenetic Nomenclature [44].

### 2.7. Doubling Time

The cells were maintained in culture, with a medium change every 2–3 days, until reaching 90% of confluence. Then, a differential trypsin treatment was performed, the trypsin was left until the feeder cells began to detach, and the supernatant was removed. Subsequently, more trypsin was added until the tissue cells detached from the flask. The cell lines were maintained in co-culture with feeder cells and without feeder cells with medium conditioned by irradiated 3T3-J2. At each passage, Trypan Blue stain 0.4% (Thermo Fisher Scientific, Waltham, MA, USA) and the Countess 3 Automated Cell Counter (Thermo Fisher Scientific, Waltham, MA, USA) were used to obtain the total number of cells. The population doubling (PD) was calculated for each passage, for each replicate, using the equation: logarithm of (final total cell number/initial cell number seeded) to base two. The cumulative PD was calculated and plotted with the days in culture. Then, using GraphPad (version 8.4.2, GraphPad Software, Boston, MA, USA), with all the replicates for each cell line, a simple linear regression was calculated, and the 95% confidence intervals were plotted with the obtained line. The doubling time was calculated from the 1/slope of this line [45,46].

### 2.8. Immunofluorescence and Microscopy

For the phenotypic characterization of the newly established cell lines, we conducted the immunofluorescence detection of the following markers: (1) epithelial origin, the pan cytokeratin antibody [AE1/AE3] (GTX75521; GeneTex; Irvine, CA, USA); (2) proliferation, the Ki67 monoclonal antibody (orb1495419; Biorbyt, Cambridge, UK); (3) and prostate-specific and cancer status, the AMACR (Alpha-methylacyl-CoA racemase; orb646140; Biorbyt, Cambridge, UK) and PSA (L106) (Prostate-Specific Antigen; orb64274; Biorbyt, Cambridge, UK) antibodies.

The cells were seeded on glass coverslips that were maintained for 48/72 h and then fixed with 4% paraformaldehyde (PFA, Thermo Fisher Scientific, Waltham, MA, USA) for 20 min. The process was then continued with permeabilization with 0.5% Triton X-100 (Merck, Darmstadt, Germany,) for 10 min, followed by blocking for 1 h with 1% bovine serum albumin (BSA, Thermo Fisher Scientific, Waltham, MA, USA). Afterwards, there was an overnight incubation with the primary antibody, followed by incubation with the secondary antibodies (Goat anti-Rabbit Alexa Fluor 594 #A11037 or Goat anti-Mouse Alexa Fluor 488 #A32723; Invitrogen, Waltham, MA, USA) for 1 h. The nucleus was stained with 4′,6-diamidino-2-phenylindole (DAPI, BioRad, Hercules, CA, USA). Lastly, the coverslips were mounted using Mowiol 4-88 (Sigma, Darmstadt, Germany) solution containing N-propyl gallate (Sigma, Darmstadt, Germany). All the immunofluorescence images were captured in the inverted Leica DMI6000 microscope, equipped with a HCX PL APO CS 63×/1.30 Glycerol objective, a Hamamatsu Flash 4.0 camera, and a motorized stage, controlled by LASX software (v3.8.1.26810).

In the particular case of Ki67, we conducted a quantification of the cells that expressed this marker. Large regions of the image were acquired using LASX Navigator for both DAPI and Ki67, with corresponding filters and exposure times of 130 ms and 190 ms, respectively. The total number of cells was determined in a semi-automated manner using Fiji/ImageJ v2.16.0/1.54p [47,48]: cells were manually identified, based on the DAPI channel, and then the “Analyze Particles” tool was used to exclude non-cellular objects and extract the total number of cells in the images (with parameters: size = 50–500 and circularity: 0.00–1.00). The total number of Ki67-positive cells within that pool was determined manually using the Fiji/ImageJ “Multi-point” tool. A total of 1000–4000 cells were counted per replicate (three replicates from distinct passages).

### 2.9. Wound Healing Assay

Cells were seeded in an Ibidi Culture-Insert 2 Well system (Ibidi, Gräfelfing, Germany) in a 24-well plate and maintained for 24 h at 37 °C and 5% CO_2_ to allow cell adhesion. Cell seeding densities were optimized for each cell line to achieve confluency (40,000 cells/well for SSA PC cell lines and 60,000 cells/well for commercial PC cell lines). Culture inserts were then removed, and cells were rinsed with PBS and incubated in medium overnight (F medium supplemented with ROCK inhibitor for PC cell lines and complete RPMI for commercial PC cell lines). During this period, wound closure was monitored by timelapse microscopy using a Leica DMI6000 Timelapse microscope (Leica, Wetzlar, Germany), with cells maintained at 37 °C and 5% CO_2_. Images were acquired every 10 min with a 10× objective under phase contrast.

### 2.10. High-Throughput Screening of Drugs

A high-throughput screening (HTS) was performed with the Prestwick Chemical Library (Prestwick Chemical Libraries, Illkirch-Graffenstaden, France) containing 1280 compounds, mainly FDA-approved. The SSA-PC01 prostate cells were seeded in 384-well plates (Corning #3570; Corning, New York, NY, USA) in F medium, at a density of 1.5 × 10^3^ cells per well, using the automated MultidropTM Combi Cell and Reagent Dispenser (Thermo Fisher Scientific, Waltham, MA, USA). Plates were incubated overnight at 37 °C, 5% CO_2_ to allow cell attachment. Then, each compound dissolved in DMSO was dispensed per well into the plates using a pintool (V&P Scientific, San Diego, CA, USA) coupled to the MDT arm of a JANUS automated liquid handler (PerkinElmer, Waltham, MA, USA), resulting in a final concentration of 4 µM. Each compound was tested in a single well per plate in the primary screen, without technical or biological replicates, in line with standard practice for large-scale primary HTS. After 48 h incubation, 12.5 µL of CellTiter-Glo Luminescent Cell Viability Assay (Promega, Madison, WI, USA) was added to each well. Plates were shaken for 10 min at 500 rpm in an orbital shaker, and RLUs (relative light units) were acquired for each well using the VICTOR Nivo Multimode Microplate Reader (Revvity, Waltham, MA, USA). Cells treated with 4 µM Doxorubicin were used as positive controls, and cells treated with 0.4% DMSO as negative controls [49,50]. Compounds inducing a reduction of at least 30% in cell viability relative to the negative control were considered hits from the HTS, and were annotated for the mechanism of action by recurring to information from the DrugBank 6.0 database [51].

A validation was conducted in the SSA-PC01 prostate cells for all hits, with each compound tested for six concentrations (16, 8, 4, 2, 1 and 0.5 µM), in duplicate. The seeding protocol, incubation times and RLU measurement method were conducted as described above. Compounds that induced a reduction of at least 75% in cell viability at the highest concentration, relative to the negative control (DMSO), were considered hits.

Some of these hits were selected (one or two hits per mechanism of action) for further half-maximum inhibitory concentration (IC_50_) determination, for all newly established PC cell lines, as well as in the two commercial PC cell lines. A total of 10 concentrations whose range depended on the individual results obtained in the validation were tested (reported in Supplementary Table S2). Each experiment was performed in three biological replicates, maintaining the same seeding density, culture conditions, and viability assay described above. Luminescence values were again normalized against the negative controls. Dose–response curves were generated and fitted using a four-parameter logistic regression model in GraphPad Prism (version 8.4.2, GraphPad Software, Boston, MA, USA) to determine IC_50_ values for each compound and cell line. The Welch *t*-test was applied to compare the IC_50_ mean values of commercial vs. established PC cell lines, using RStudio v2026.05.

## 3. Results

### 3.1. Establishment of the SSA-PC Cell Lines

**Figure 1:**
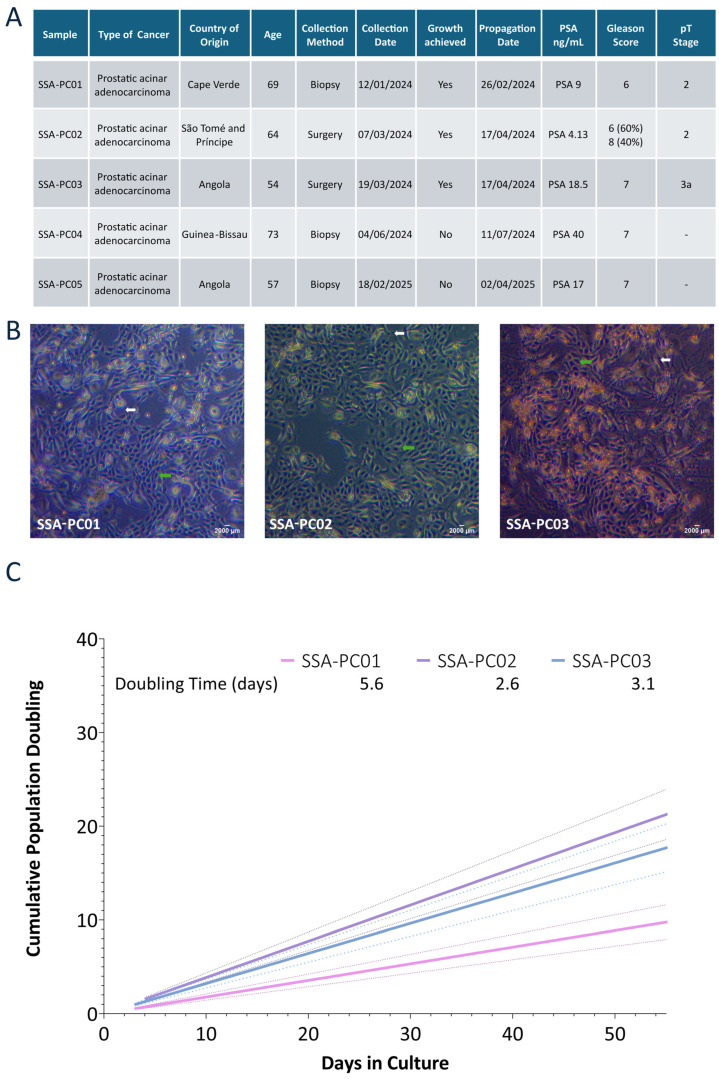
The PC cell lines. (**A**) Characteristics of the collected samples (Collection Date indicates the date of surgery/biopsy; Propagation Date indicates the date when samples were thawed, processed, and placed into culture; pT refers to the primary tumour category of the AJCC pTNM staging). (**B**) Bright-field microscopy images of the established cell lines, with white arrows indicating the feeder cells and the green arrows indicating the epithelial cells. Images were captured with Zeiss Primovert—Axio Cam ERc C5s (Zeiss, Oberkochen, Germany), at 10× objective. (**C**) Cumulative population doubling for the established cell lines. Each line represents the linear regression of the cumulative PD for each cell line, with the 95% confidence interval represented in dotted lines. Also indicated are the doubling time (days) for each established cell line.

The five individuals (Figure 1A) were diagnosed at ages in the range 54–73 years with prostatic acinar adenocarcinoma. The Gleason scores varied between low (6) and intermediate (7), with SSA-PC02 having a significant tumour proportion (40%) with a high (8) score. The PSA values at diagnosis ranged from 4.13 to 40 ng/mL.

The propagation by CR was successful for three samples, SSA-PC01, SSA-PC02 and SSA-PC03. This corresponds to a success rate of 60%. The unsuccessful cases were due to no growth (SSA-PC05) and contamination with another cultured sample during the laboratory processing (SSA-PC04). The time since collection/freezing and initial propagation was around one and a half months for all cases, so this factor did not influence the success of culture growth.

The growth of the successful cell lines was monitored by bright-field microscopy (Figure 1B). The feeder cells were elongated (fibroblastic shape) in comparison with the propagated cells, which were smaller and more rounded (epithelial shape). Epithelial colonies could be observed at around 5–7 days, and they proliferated to reach confluence in approximately 7–12 days. The growth of the prostate cells was rapid, with SSA-PC01 having a doubling time of 5.6 days, SSA-PC02 2.6 days and SSA-PC03 3.1 days (Figure 1C). The maximum durations of continuous culture for each sample (Supplementary Figure S1), respectively, were 56 days with eight cumulative population doublings, 102 days with 37 cumulative population doublings, and 70 days with 20 cumulative population doublings. In a similar way, when low-passage cryovials were thawed, the cells reached confluence within approximately two weeks, supporting the long-term viability of the banked stocks.

### 3.2. Molecular Characterisation of the PC Cell Lines

**Figure 2:**
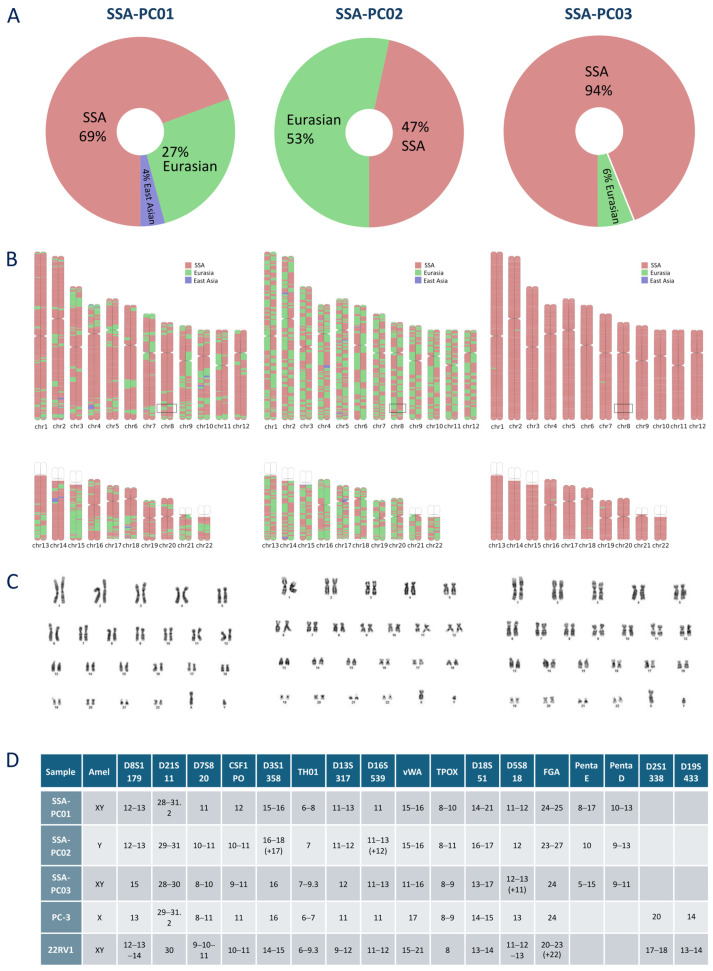
Molecular characterisation of the PC cell lines. (**A**) Ancestry admixture of the collected samples for three K backgrounds (SSA, Eurasian and East Asian). (**B**) Admixture mapping along the genome for the three parental ancestries, with a square on chromosome 8 highlighting the PC-associated 8q24.21 region. (**C**) Karyotypes in metaphase G-banding. (**D**) STR profiles for the established and commercial cell lines (minor alleles with a signal between 10 and 20% of the major allele are indicated with “+”).

We ascertained the genetic ancestry of the SSA-PC cell lines by using a DNA array and the ADMIXTURE algorithm (Figure 2A). SSA-PC01 was derived from a Cape Verdean national with a 69% SSA background admixed with 27% Eurasian and 4% East Asian. SSA-PC02, from São Tomé and Príncipe, had a 53% Eurasian and 47% SSA admixture. SSA-PC03, from Angola, had a 94% SSA and 6% Eurasian background. The mtDNA haplogroup affiliation for all the samples was SSA ancestry (SSA-PC01: L3b; SSA-PC02: L1c2b1; SSA-PC03: L3f1), while for the paternal side, the two individuals with a higher Eurasian admixture had Eurasian Y-chromosome haplogroups (SSA-PC01: R1b1a2a1a2; SSA-PC02: R1b1a2a1a2c1a1a1) and the other had an SSA haplogroup (SSA-PC03: E1b1a1a1d1). We also conducted admixture mapping, which allows the visualisation of the ancestry admixture along the chromosomes (Figure 2B), informing us that SSA-PC02 (~50:50 Eurasian to SSA admixture) is not a first-generation admixed individual (short ancestry fragments).

The genomic profiles also allow us to provide some insights into known SSA-PC-associated variants/genomic regions, despite the big gaps in GWAS cataloguing for this ancestry. The admixture mapping allowed us to confirm that in relation to the PC-associated 8q24.21 region, the three individuals have 100% SSA ancestry for the segment 129,001,918–129,678,850 bp (GRCh38.p14), containing *CCDC26* and miRNA-3686 genes (highlighted in Figure 2B; Supplementary Table S1). The PRS estimates (Supplementary Figure S2) show that SSA-PC01 has a lower value than the mean of the male Yoruba population, while SSA-PC02 and SSA-PC03 have a similar and high PRS in the Q4 (75–100%) of the reference population.

Regarding the karyotype characterization, all the established SSA-PC cell lines have a normal karyotype, and the male sex was confirmed (Figure 2C). This characterisation was conducted in cells at low passages, and indicates that the CR method did not introduce chromosomal abnormalities.

The STR characterisation was conducted in the three SSA-PC and two commercial PC cell lines (Figure 2D). Mining the CLASTR 1.4.4 database allowed us to ascertain that the profiles for the three SSA-PC cell lines were new, not matching any commercial cell line, ensuring that there was no cross-contamination in the lab. For the two commercial lines, the obtained profiles matched the registered profiles in the database. The amelogenin marker confirmed the presence of the Y-chromosome in all the samples, except in the commercial line PC-3, which is in accordance with data available in Cellosaurus. The non-detection of the X-chromosome marker in SSA-PC02 could be due to diversity in the primers included in the commercial STR kit, as described before [52].

### 3.3. Phenotyping of the PC Cell Lines

**Figure 3:**
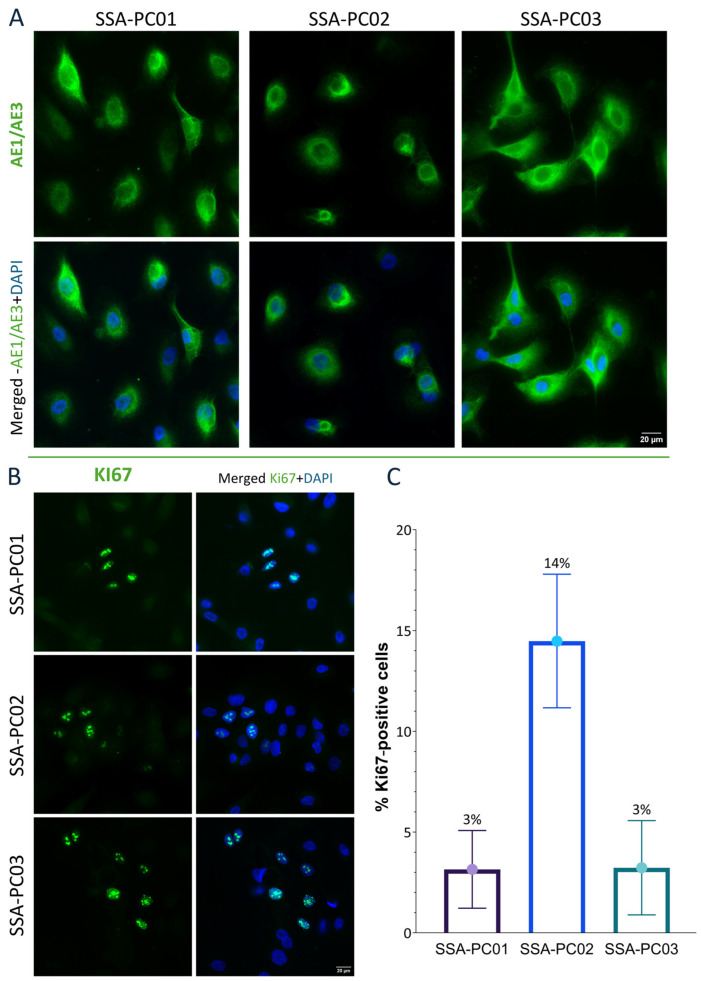
Immunofluorescence assessment of epithelial origin and proliferation in the established PC cell lines. Immunofluorescence staining for cytoplasmic AE1/AE3 (**A**) and nuclear Ki67 markers ((**B**), green), visualized with the inverted Leica DMI6000 microscope. Nuclei were stained with DAPI (blue). The scale bar corresponds to 20 μm and images were obtained with a 63× objective. (**C**) Graph of the percentage of Ki67-positive cells for the established PC cell lines.

To confirm that the proliferating cells were of epithelial origin, immunofluorescence was conducted for the AE1/AE3 marker (Figure 3A), which identifies all normal and tumour epithelial cells [53]. All SSA-PC cells are positive for this marker in the cytoplasm.

As a proliferative marker, Ki67 staining of nuclei was used (Figure 3B). SSA-PC02 displayed a mean of 14% Ki67-positive cells, while the other two SSA-PC cell lines had a mean value of 3% (Figure 3C). Values higher than 10% are rare in PC, and are usually considered indicative of aggressive cases [54].

**Figure 4:**
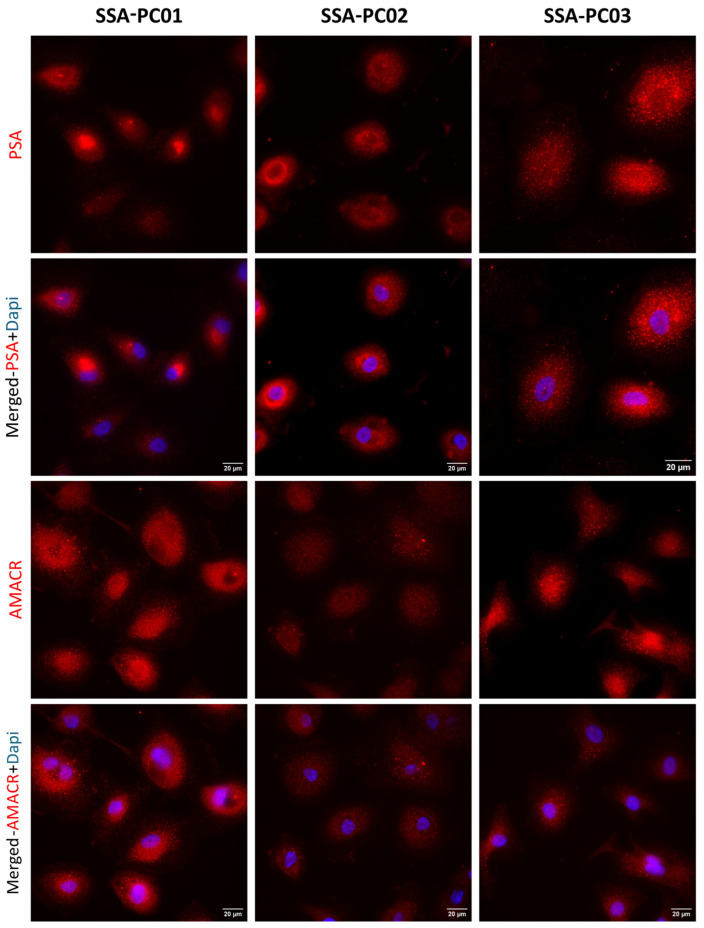
Immunofluorescence assessment of prostate origin in the established PC cell lines, for cytoplasmatic AMACR and PSA markers (red), visualized with the inverted Leica DMI6000 microscope. Nuclei were stained with DAPI (blue). The scale bar corresponds to 20 µm and images were obtained with a 63× objective.

In order to ascertain the tissue of origin, the prostate-specific antibodies AMACR and PSA were also assessed (Figure 4; negative and positive controls are displayed in Supplementary Figure S3). PSA is an androgen-regulated serine protease with high specificity for prostatic tissue, being a biomarker for PC diagnosis [54,55]. As expected, PSA was present in all SSA-PC cell lines. AMACR, an enzyme overexpressed in the majority of PC cases [56], was also positive in all SSA-PC cell lines.

**Figure 5:**
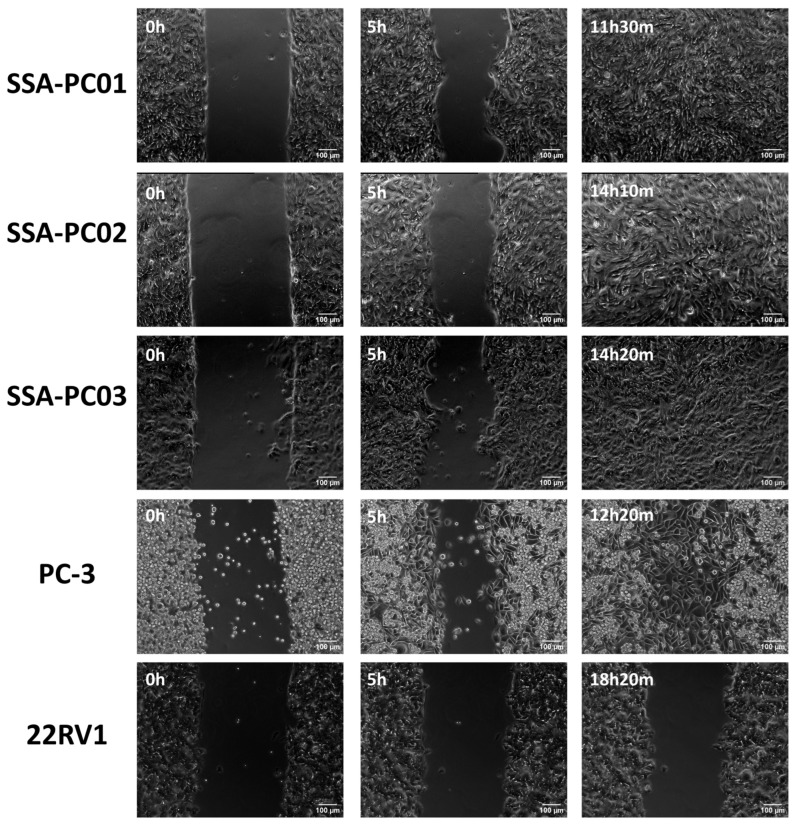
Wound healing assay. Representative images of the wound closure, at two static (0 h and 5 h) and at the closure/limit-of-experiment timepoints.

To assess the ability of the PC cell lines to migrate, wound healing assays were performed (Figure 5). The established cell lines were able to repair the wound in the time range between 11 h and 14 h 30 min, as was the commercial cell line PC-3 (in 12 h 20 min). The commercial cell line 22RV1 was not able to repair the wound.

### 3.4. Drug High-Throughput Screening

**Figure 6:**
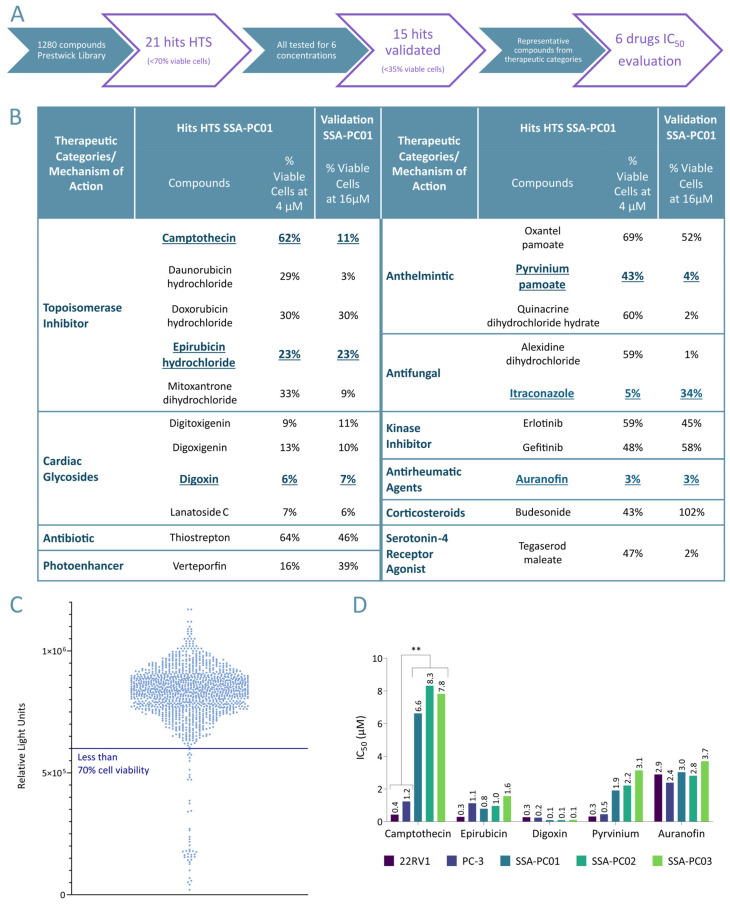
High-throughput screening (HTS) of drugs in the PC cell lines. (**A**) Schematic representation of the drug screening. (**B**) Hits for the HTS and the validation conducted with SSA-PC01. Therapeutic categories or mechanism of action according to DrugBank are indicated. Drugs in bold and underlined were chosen for IC_50_ evaluation. (**C**) Relative light unit (RLU) values for the 1280 compounds from the Prestwick library and controls. The line represents the threshold for 70% cell viability. (**D**) IC_50_ values for five selected drugs in the three established and two commercial PC cell lines. ** indicates a significant Welch *t*-test adjusted *p*-value.

Patient-derived cancer cell lines offer the advantage of enabling HTS (Figure 6A). The 1280 compounds contained in the Prestwick Chemical Library were tested in a high-throughput screening with the SSA-PC01 cell line. After normalization for the solvent (DMSO), a total of 21 drugs led to a reduction of at least 30% cell viability (or a maximum of 70% cell viability; Figure 6B,C; Supplementary Table S2). These hits were further analysed in a validation test with a range of drug concentrations, and 15 hits passed a stricter threshold of a reduction of at least 65% of cell viability for the highest concentration (16 µM; Figure 6B; Supplementary Table S3), representing six therapeutic categories/mechanisms of action: topoisomerase inhibitors; cardiac glycosides; anthelmintics; antifungal agents; antirheumatic agents; and serotonin-4 receptor agonists. A total of six drugs were not validated under this stricter threshold, namely the antibiotic thiostrepton, photoenhancer verteporfin, anthelmintic oxantel pamoate, kinase inhibitors erlotinib and gefitinib, and corticosteroid budesonide.

We then selected some drugs from each of the six validated therapeutic categories/mechanisms of action for an IC_50_ assay. For the inhibitors of topoisomerases, we selected one for each topoisomerase type 1 (TOP1; the only validated hit in this category was camptothecin) and type 2 (TOP2; epirubicin hydrochloride). Other TOP2 inhibitors were not prioritized for the following reasons: doxorubicin was included as a positive control in the HTS (following the literature; e.g., [57,58]); mitoxantrone has been reported to exhibit a higher risk of cardiotoxicity compared with epirubicin [59]; daunorubicin is a first-generation anthracycline, whereas epirubicin is a second-generation analogue developed to improve the therapeutic index, including reduced cardiotoxicity and a more favourable pharmacokinetic profile [60]. For cardiac glycosides, we selected the FDA-approved digoxin, while the remaining ones are non-FDA-approved compounds (https://www.accessdata.fda.gov/scripts/cder/daf/index.cfm, consulted on 23 December 2025). Within the anthelmintic category, pyrvinium pamoate was selected for its reported antitumor activity in PC models [61]. While anthelmintic oxantel pamoate displayed low cytotoxicity in the primary screen and validation (69% and 52%, respectively), quinacrine dihydrochloride hydrate has reported limited preclinical evidence of efficacy in PC [62,63], being mainly used in combination regimens [64]. In the antifungal category, itraconazole was selected over alexidine, due to its documented anticancer activity [65,66]. For the antirheumatic drug category, auranofin was the only identified hit. The serotonin-4 receptor agonist tegaserod maleate was not selected, because its safety and effectiveness have not been established in men, and it was even withdrawn from the market in the past following reports of increased cardiovascular ischemic events in women, raising additional safety concerns [67,68].

The results for the IC_50_ assay (Figure 6D; Supplementary Figure S4) showed that: (1) the inhibitor of TOP2 epirubicin hydrochloride seems to be more efficient than the inhibitor for TOP1 camptothecin for all PC cell lines and especially so for the established SSA ones (IC_50_ values for camptothecin are statistically significantly higher in SSA-PC cell lines in relation to the values for the commercial ones; Welch *t*-test adjusted *p*-value = 0.009966; Supplementary Table S5); (2) the cardiac glycoside digoxin is equally efficient for all the PC cell lines; (3) the anthelmintic pyrvinium is more efficient for the commercial cell lines than the established ones (statistically non-significant for adjusted *p*-value = 0.064233); (4) the antirheumatic agent auranofin is also equally efficient for all PC cell lines; and (5) the antifungal itraconazole never killed more than 50% of the cells in all the tested concentrations (the range of concentrations was even higher than for the ones tested for the other drugs, and a further concentration increase was not feasible due to limitations imposed by the required DMSO concentration, which would have exceeded acceptable solvent control thresholds; not included in Figure 6D).

We queried the ClinPGx database (https://www.clinpgx.org/; accessed on 14 July 2026) to identify pharmacogenomic variants that might explain the differential sensitivity to camptothecin between SSA and EUR cell lines. No variants associated with camptothecin were reported. However, this may reflect a bias due to the preferential clinical use of its semisynthetic derivatives, irinotecan and topotecan, rather than camptothecin itself. These derivatives are included in the library that was used here, but they were not validated because they were above the threshold for a hit in the first step (although topotecan was very close to the threshold, with 78% of cell viability; irinotecan 86%). ClinPGx lists several pharmacogenomic variants associated with irinotecan, especially in genes involved in its metabolism, such as the *UGT1A1* gene (highest evidence of association with dosage and toxicity), and in its transport, such as the ABC (ATP-binding cassette) gene family.

Unexpectedly, drugs present in the Prestwick Chemical Library that are FDA-approved for PC treatment (https://www.cancer.gov/about-Cancer/treatment/drugs/prostate, accessed on 28 December 2025; Supplementary Table S4) were not hits in the HTS. Specifically, the library includes the androgen receptor inhibitors flutamide, bicalutamide, and nilutamide (percentage of cell viability in the HTS was 100%), the microtubule inhibitor docetaxel (percentage of cell viability in the HTS—100%). The only exception being the already mentioned TOP2 inhibitor mitoxantrone dihydrochloride, with a 33% cell viability, also a FDA-approved drug for PC treatment.

## 4. Discussion

The CR method is relatively recent and characterized by high efficiency (reported as high as 90% [29]) and a short time for the establishment of primary cell lines. In this study, the CR success rate for the patient-derived PC samples was 60%, leading to the establishment of three SSA-PC cell lines. Initial growth was also observed in another sample (SSA-PC04); however, subsequent STR profiling revealed cross-contamination with another established cell line, illustrating the necessity of routine genetic validation during cell propagation. The fact that most of the available samples were obtained from biopsy, limiting the amount of available tissue, could have impacted the lower success rate in this study compared with reported ones. The new SSA-PC cell lines (SSA-PC01, SSA-PC02 and SSA-PC03) were maintained in continuous culture for 56, 102 and 70 days, respectively, before proliferative capacity declined. During this period, multiple cryovials were generated at different passages to ensure sufficient material for comprehensive characterization, long-term storage, and future studies using this novel SSA-PC cell line panel. It is our aim to share this panel with a recognized repository and, meanwhile, it is available upon contact with colleagues conducting oncobiology research focused on SSA ancestry.

Cell proliferation of the new SSA-PC cell lines occurred in the order of days (SSA-PC01: 5.6 days, SSA-PC02: 2.6 days, SSA-PC03: 3.1 days), slightly higher than for commercial PC cell lines (the Cellosauros database [69] reports duplication times of 1–2 days for PC-3 and 1–4 days for 22RV1). This difference is expected, as the long-established immortalized PC-3 and 22RV1 cell lines have likely accumulated a higher mutational burden.

The Ki67 proliferation index is a widely used marker of cellular proliferation across multiple cancer types. In PC, the definition of low and high Ki67 expression remains heterogeneous, with reported cut-off values for low versus high proliferation ranging between 1.4% and 10% [70,71]. Taking this variability into account, and following others [70], we defined high Ki67 expression as values above 5%. Moreover, elevated Ki67 expression has been associated with aggressive tumour behaviour, increased risk of metastatic progression [71], and higher Gleason grade (>10% Ki67 staining related to >7 Gleason grade) [72]. Beyond its role as a prognostic indicator, Ki67 has been shown to provide additional information that may support clinical decision-making, including guiding neoadjuvant treatment strategies and predicting biochemical recurrence following radical prostatectomy, particularly in patients with otherwise favourable pathological features [73]. Within this context, two of the SSA-PC cell lines exhibited low Ki67 levels (3% for both SSA-PC01 and SSA-PC03), while SSA-PC02 showed a high Ki67 level (14%). This finding of a high proliferation for SSA-PC02 is consistent with its shortest doubling time (2.6 days) and longest uninterrupted period in culture (102 days) among the new SSA-PC cell lines. Although SSA-PC02 was classified as Gleason grade 6, pathological anatomy analysis revealed that approximately 40% of the analysed tumour exhibited Gleason grade 8. It is possible that the Gleason grade 8 population outgrew the 6 during culture establishment, becoming predominant in the SSA-PC02 cell line.

In the wound healing assay, all SSA-PC cell lines were able to close the wound within 18 h, testifying to their migratory capacity. Despite the higher proliferative activity for SSA-PC02, this cell line was not the fastest in repairing the wound, indicating that high proliferation does not linearly correlate with enhanced migratory capacity, as was also observed in a gastric cancer cell line [74]. For the commercial PC cell lines, 22RV1 was not able to close the wound, an observation that matches previous reports that 22RV1 cells are unable to fully close a wound even after 48 h [75], with authors suggesting that its non-metastatic origin is associated with limited migratory capacity [76]. In contrast, PC-3 cells have been reported to achieve wound closure within approximately 12 h [77], which aligns with the results obtained in our experiments.

The detection of the AE1/AE3 antibody for the majority of cells in the SSA-PC cell lines confirmed their epithelial identity, rather than contaminating components. In fact, stromal and fibroblast populations (originating from the patient tissue) may persist and overgrow the epithelial cells [29] during culturing, but this was not the case in this study. PSA, a protein produced by the cells of the prostate gland, is widely used clinically as a serum biomarker of prostate tissue activity, with overexpression indicating prostate adenocarcinoma or several benign conditions, such as, for example, hyperplasia [78]. The expression of this prostate-specific protein in the SSA-PC cell lines showed the preservation of the prostate-specific molecular features during CR propagation. In parallel, the overexpression of AMACR is detected in most cases of PC (around 80–90%), being considered a sensitive biomarker of PC tumour tissues [79,80,81]. The AMACR positivity observed in the SSA-PC cell lines confirmed their malignant nature. Together, AE1/AE3, PSA and AMACR give strong evidence of the PC-specific epithelial and malignant identity of the established SSA-PC cell lines. The karyotype analysis further confirmed that no large chromosome structural mutations were introduced with the CR propagation.

The genetic ancestry analysis confirmed that the established PC cell lines had at least half SSA background, with uniparental markers (mtDNA and Y-chromosome) allowing us to confirm that the European admixture in SSA-PC01 and SSA-PC02 was of paternal origin. This heterogeneity in EUR admixture (6%, 26% and 53%) provides a valuable resource for investigating the contribution of SSA ancestry to PC. In fact, the admixture mapping allowed us to confirm that the previously PC + SSA-associated 8q24.21 region was 100% from the SSA background in the three SSA-PC samples, despite its heterogeneity in the EUR admixture. This region contains the RNA coding genes *CCDC26* (long noncoding RNA) and miRNA-3686. *CCDC26* has been associated with various cancer types: acute myeloid leukaemia, where the gene overexpression is thought to regulate expression of the tyrosine kinase receptor *KIT* [82]; genetic risk for glioma [83,84]; and in pancreatic cancer, where its expression was positively correlated with *PCNA* and *Bcl2* [85]. miRNA-3686 has also been associated with cancer, namely in the pancreas where it seems to regulate the polo-like kinase 1 (*PLK* [86]). This region is adjacent to the region containing *MYC* and *PVT1* genes, but a link between *CCDC26* and these genes has not been established [87]. The panel of SSA-PC cell lines established here, together with the development of additional SSA-PC cell lines, will be essential for functionally characterizing the role of the 8q24.21 region in PC. Also, the estimated PRSs based on Janivara et al.’s [23] GWASs, with the largest SSA-PC cohort characterized so far, indicated that SSA-PC02 and SSA-PC03 had high values, in the Q4 (75–100%) of an SSA reference population, while SSA-PC01 had a low value, below the mean of the reference population. These values should be interpreted with caution, as they may be affected by limitations in imputation and the currently explained heritability. Nevertheless, they are consistent with the more aggressive tumour phenotypes observed in SSA-PC02 and SSA-PC03.

The HTS conducted with the 1280 compounds contained in the Prestwick Chemical Library led to 15 validated hits with restricting cut-offs (less than 35% of viable cells), belonging to six therapeutic categories/mechanisms of action, TOP1/2 inhibitors, cardiac glycosides, anthelmintics, antifungals, antirheumatic agents and serotonin-4 receptor agonists. This diversity is interesting to explore.

The class of TOP inhibitors was the most identified in the HTS. One of the three TOP1 inhibitors included in the library was classified as a hit (camptothecin), whereas topotecan (78% cell viability) and irinotecan (86%) were not. In contrast, four of the six TOP2 inhibitors were hits, with only etoposide (94% cell viability) and dexrazoxane hydrochloride (100%) failing to meet the hit criteria. Among the compounds selected for validation, both the TOP1 inhibitor camptothecin and TOP2 inhibitor epirubicin hydrochloride markedly reduced cell viability (11% and 23%, respectively). However, IC_50_ analysis revealed that epirubicin was more efficient than camptothecin in the SSA-PC cell lines. Notably, SSA-PC cell lines were approximately ninefold less sensitive to camptothecin than the commercial PC cell lines. Several key mechanistic differences between TOP1 and TOP2 inhibitors warrant consideration when interpreting these findings. TOP2 inhibition induces direct double-strand DNA breaks, which are generally considered more cytotoxic than the single-strand DNA lesions generated by TOP1 inhibitors [88,89]. In PC, TOP2 also contributes to androgen receptor-dependent transcription, and its inhibition may therefore disrupt key oncogenic transcription programs, in addition to inducing DNA damage [90,91]. In addition, anthracyclines such as epirubicin generate reactive oxygen species [92,93], leading to oxidative DNA damage that can occur independently of DNA replication. Further analyses are needed to shed light on the greater efficacy of TOP2 inhibition observed in PC models across ancestries.

TOP inhibitors represent a promising therapeutic class, particularly in *MYC*-overexpressing cancers [94]. This may be especially relevant in prostate tumours from SSA patients, which frequently exhibit *MYC* overexpression. An MYC-nucleated “topoisome” complex has been identified that recruits both TOP1 and TOP2 to promoters, gene bodies, and enhancers, increasing activity [95]. Because African ancestry-specific risk variants at the 8q24 locus may drive this persistent *MYC* hypertranscription, SSA PC cells may adapt to this continuous stress by upregulating TOP1 turnover or clearance pathways, thereby reducing their sensitivity to TOP1 inhibitors. This hypothesis warrants further investigation.

Despite their therapeutic potential, TOP-inhibitor-based therapies have shown limited efficacy (e.g., 13–32% response rates in colorectal cancer [96]) and have been associated with mechanisms of resistance that remain incompletely understood [97]. Pharmacogenomic differences may also contribute to inter-ancestry variation in treatment response. For example, variants in *UGT1A1*, a key determinant of irinotecan metabolism, influence TOP1 inhibitor dosing. Taylor et al. [98] identified ancestry differences in the regulatory architecture of *UGT1A1* between AA and EA individuals, suggesting that additional cis- and/or trans-acting regulatory factors affecting *UGT1A1* expression remain to be identified in populations of African ancestry.

Mitoxantrone hydrochloride, the FDA-approved TOP2 inhibitor for PC treatment, is associated with a high risk of cardiotoxicity [59]. Therefore, the strong activity of epirubicin observed in this study suggests that it may represent an alternative TOP2-targeting strategy worthy of further investigation. The availability of our SSA-PC cell line panel provides a unique platform to dissect the biological basis underlying the differential responses to TOP1 and TOP2 inhibitors in PC in patients of SSA ancestry.

The cardiac glycoside category, usually prescribed for the treatment of heart failure, also had four hits in the HTS, which represent all the compounds included in the library. Cardiac glycosides are inhibitors of the Na^+^/K^+^-ATPase, but their activity as antineoplastic agents seems to be variable [99], involving mechanisms that range from regulation of oncogenes [100] to a possible influence on DNA damage repair regulatory proteins [101]. Digoxin, in this category, was the compound that exhibited the lowest IC_50_ for the six tested drugs in all the PC cell lines. This result is consistent with previous reports [102], where the mean IC_50_ across six commercially available PC cell lines was 0.163 µM. Moreover, in PC, the calcium channels that digoxin inhibits seem to regulate the proliferation of PC cells [103,104]. Notably, epidemiological studies have also suggested that the clinical use of digoxin for cardiac reasons (especially in patients taking it for more than 10 years) is associated with an approximately 25% reduced risk of PC, further supporting its potential relevance in this disease context [102]. A PC clinical trial with digoxin was conducted [105], leading to the observation that 38% of the patients had a decrease in PSA doubling time.

For the anthelmintic compounds, the library included over 20 compounds, and three were hits in the HTS (pyrvinium pamoate and quinacrine dihydrochloride hydrate more efficiently than oxantel pamoate). In the case of anthelmintic pyrvinium pamoate tested for the IC_50_, all SSA-PC cell lines displayed IC_50_ values at least twofold higher than PC-3, for which an IC_50_ of approximately 0.5 µM was obtained. This value is consistent with previously reported IC_50_ values for pyrvinium in PC-3 cells (0.7145 µM [106]). Pyrvinium is known to target the endogenous androgen receptor and seems to have considerable therapeutic potential to treat castration-resistant PC [107]. Although we did not perform IC_50_ for quinacrine dihydrochloride hydrate, this compound also was shown to be very efficient against cell viability in the validation test at 16 µM, and it has been explored in the past as a combined therapy for PC with paclitaxel [63].

For the antirheumatic agent auranofin, the IC_50_ value obtained in this study for PC-3 (2.4 µM) is concordant with a value of 2.5 µM, in an assay with 24 h of treatment [108], compared with 48 h in our assay. This reassures us in concluding that this agent is efficient in all the SSA-PC and commercial PC cell lines. Auranofin is a gold compound that acts as a pro-oxidant agent through inhibition of thioredoxin reductase and has been extensively investigated as a candidate for drug repurposing in cancer, including in pancreatic, breast and lung cancer [109].

The list of FDA-approved drugs for PC treatment includes 23 drugs, of which only five are included in the Prestwick Chemical Library. Curiously, the three androgen receptor inhibitors (flutamide, nilutamide and bicalutamide) and the microtubule inhibitor (docetaxel) were not able to impact cell viability in the HTS, and only the already mentioned TOP2 inhibitor (mitoxanthrone hydrochloride) did so. Androgens play a central role in regulating proliferation and cell growth in prostate tissue, in both normal and malignant contexts [110]. Antiandrogens primarily inhibit androgen receptor-driven proliferation and cell cycle progression [111], but do not typically induce rapid or acute cell death. The drug concentration used in the HTS (4 µM) may represent a limiting factor for the detection of activity for the three antiandrogens present in the library, as previous studies reported higher IC_50_ concentrations: exceeding 11 µM for flutamide [112] and 45 µM for bicalutamide in PC cell lines [113]; for nilutamide, no values have been reported in PC, but studies in breast cancer models report IC_50_ values exceeding 150 µM [114]. Docetaxel is a microtubule-stabilizing agent that induces cell cycle arrest and apoptosis primarily during the G2/M phase [115,116]. Reported IC_50_ values for docetaxel in PC cell lines show that it is effective in the nanomolar range, for example, ranging from 25 nM [117] to 10 nM [118] for PC-3. The fact that we did not detect this drug as a hit in our HTS could be related to the experimental setup, in which each drug is added to only one well, without any replicates, which could sometimes lead to false negatives or positives.

Our results also provide insights into kinase inhibitors, which were hits in the HTS, but did not pass the more restrictive threshold in the validation test. Their effect is primarily cytostatic rather than cytotoxic, resulting in growth inhibition without the induction of acute cell death [119]. This class of drugs is not a typical clinical indication for PC (not FDA-approved for PC), and the two epidermal growth factor receptor (EGFR) kinase inhibitors present in the library, erlotinib and gefitinib, have consistently shown limited efficacy in PC models [120,121]. EGFR overexpression seems to be a biomarker of PC dissemination to rigid organs, preferentially bones [122], not of primary tumours.

The methods used in this study have several limitations. The panel contains three successfully established SSA-derived cell lines from an initial cohort of five, reflecting the technical challenges of primary tissue culture. As with other cell propagation methods, CR involves some technical considerations worth noting. Feeder cells require effective growth arrest to prevent overgrowth, but irradiation issues may not result in complete growth arrest. The impact of this artefact can be overcome by recurring to conditioned medium. The ROCK inhibitor that is used throughout culture may influence cytoskeletal dynamics and affect migration and invasion readouts. However, Liu et al. [29] reported no effect of ROCK inhibition on these parameters, and our findings similarly show that the SSA-PC cell lines retained the ability to repair wounds. Genomic and phenotypic drift over passaging is also a general consideration for any propagation methodology, but CR maintains greater genetic and phenotypic fidelity by not recurring to exogenous oncogenic drivers. When using an extended drug library, the HTS is commonly performed at a single drug concentration without replicates, which may lead to false negatives for compounds requiring different dosing or exposure times. If the HTS is followed by IC_50_ determination for top hits, at least this analysis will identify false positives. Finally, establishing SSA-representative preclinical models remains constrained by practical and ethical challenges in accessing tumour tissue in the African continent, including limited biobanking and cell culture infrastructures and the need for community-engaged consent processes. We overcome this challenge by collecting samples from SSA patients being treated in Portugal.

## 5. Conclusions

This study establishes and characterizes a novel panel of PC cell lines derived from patients of SSA ancestry using CR, and demonstrates their utility for drug HTS. The SSA-derived models displayed doubling times in the range 2.6–5.6 days, preserved epithelial and prostate tumour features, had a normal 46-chromosome karyotype, and were able to repair wounds. STR profiling confirmed their uniqueness and ~1 million SNP profiling confirmed at least half SSA background, with 100% SSA ancestry in parts of the PC + SSA-associated 8q24.21 region. Although the HTS approach is limited by the use of a 48 h viability assay and a fixed drug concentration (4 µM), which may reduce efficacy for some compounds, including standard-of-care androgen receptor inhibitors, it still reveals multiple opportunities for drug repurposing in PC. Drugs of the classes TOP2 inhibitors and cardiac glycosides seem to be particularly efficient. The new finding of SSA-associated lower efficiency of TOP1 versus TOP2 inhibitors deserves further investigation, now that we have SSA-PC cell lines available. Our results support the possible replacement of the standard-of-care drug mitoxantrone by another TOP2 inhibitor, such as epirubicin hydrochloride, given that the first has a high dose-dependent cardiotoxicity. Future multi-omics characterization of these novel SSA-PC cell lines could advance our understanding of the molecular basis of these therapeutic opportunities, ultimately supporting the development of more effective treatments for PC in individuals of SSA ancestry.

## Data Availability

The profiles for the DNA microarray can be downloaded from this link: https://www.i3s.up.pt/SSAPC/SSAPC.zip.

## Supplementary Materials

The following supporting information can be downloaded at https://www.mdpi.com/article/10.3390/cancers18152452/s1, Figure S1: Cumulative population doubling for the three established cell lines. All replicates for each cell line are represented (n1–n6). The line represents the linear regression of the cumulative PD for each cell line, with the 95% confidence interval represented in dotted lines; Figure S2: Density curve for the polygenic risk scores (PRSs) based on Janivara et al. [23] for the Yoruba (YRI) male population from the 1kGP database and for the three patients from whom the SSA-PC cell lines were established; Figure S3: Immunofluorescence controls for cytoplasmatic AMACR and PSA markers (both in red). (A) Negative controls for the SSA-PC cancer cell lines (secondary-antibody-only controls); (B) Positive controls for PSA and AMCR using the cell line 22RV1. All visualized with the inverted Leica DMI6000 microscope. Nuclei were stained with DAPI (blue); Figure S4: IC50 calculation for six drugs in five cell lines. Cell viability across the ten different concentrations for the six drugs tested. Each cell line is represented with a different symbol and colour. Each point represents the mean of the three replicates. All standard deviations are also represented; Table S1: RFMix inference of ancestry in the 8q24.21 region. The suffixes “.0” and “.1” in front of each cell line refer to the pair of homologous chromosomes; Table S2: Cell viability for the 1280 compounds in the primary screen; Table S3: Cell viability obtained in the validation for the 21 compounds tested for the six different concentrations; Table S4: FDA-approved drugs for prostate cancer. For the ones present in the Prestwick library, we indicate the cell viability percentage obtained in the primary screening; Table S5: Results of the Welch *t*-test comparing the IC50 values between SSA and EUR PC-CCLs.

## Abbreviations

The following abbreviations are used in this manuscript:

AA	African American
BSA	Bovine serum albumin
CCLE	Cancer Cell Line Encyclopedia
CR	Conditional reprogramming
EA	European American
GMS	Global mutational subtype
GWASs	Genome-wide association studies
H3Africa	The Human Heredity and Health in Africa consortium
HICs	High-income countries
HTS	High-throughput screen
IC50	Half-maximal inhibitory concentration
iPSCs	Induced pluripotent stem cells
PALOP	Portuguese-speaking African Countries
PC	Prostate cancer
PD	Population doubling
PFA	Paraformaldehyde
PMDA	Precision Medicine Diversity array
RLU	Relative light unit
ROCK	Rho kinase inhibitor
SNP	Single-Nucleotide Polymorphism
SSA	Sub-Saharan African
STR	Short tandem repeat
TCGA	The Cancer Genome Atlas
TOP1	Topoisomerase type 1
TOP2	Topoisomerase type 2
WGS	Whole-genome sequencing
