# Supplementary material for: Sub-Saharan African Prostate Cancer Patient-Derived Cell Lines and High-Throughput Drug Screening: Addressing Ancestry Underrepresentation in Oncobiology Research
Source: Cancers (Basel). 2026 Jul 30;18(15):2452. doi: 10.3390/cancers18152452 (PMC13465145; doi:10.3390/cancers18152452)
Supplement: Supplementary file 1 [file cancers-18-02452-s001.zip › cancers-4416503-supplementary figures.pdf]

Supplementary figures

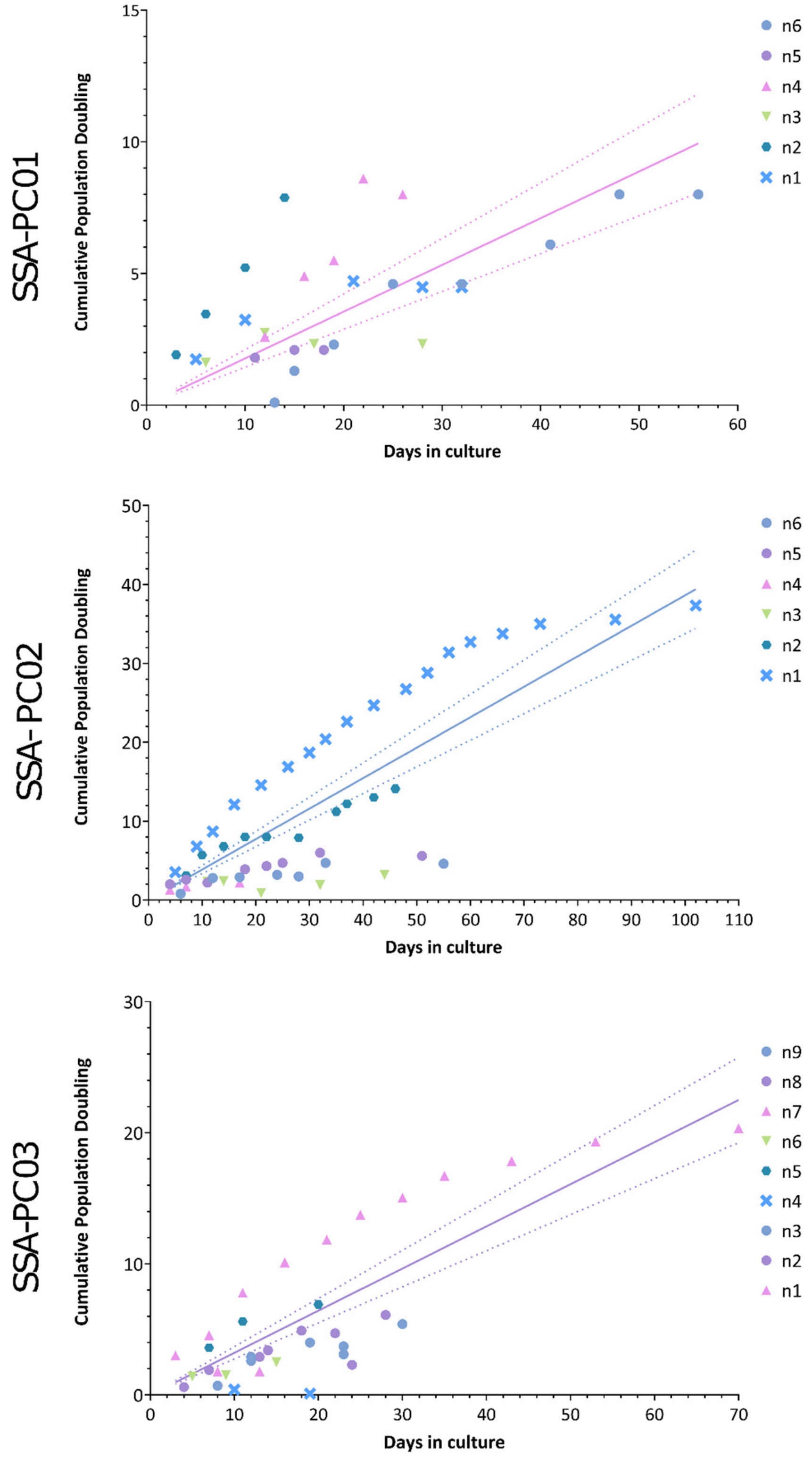

Supplementary Figure S1. Cumulative population doubling for the three established cell lines. All replicates for each cell line are represented (n1–n6). The line represents the linear regression of the cumulative PD for each cell line, with the 95% confidence interval represented in dotted lines.

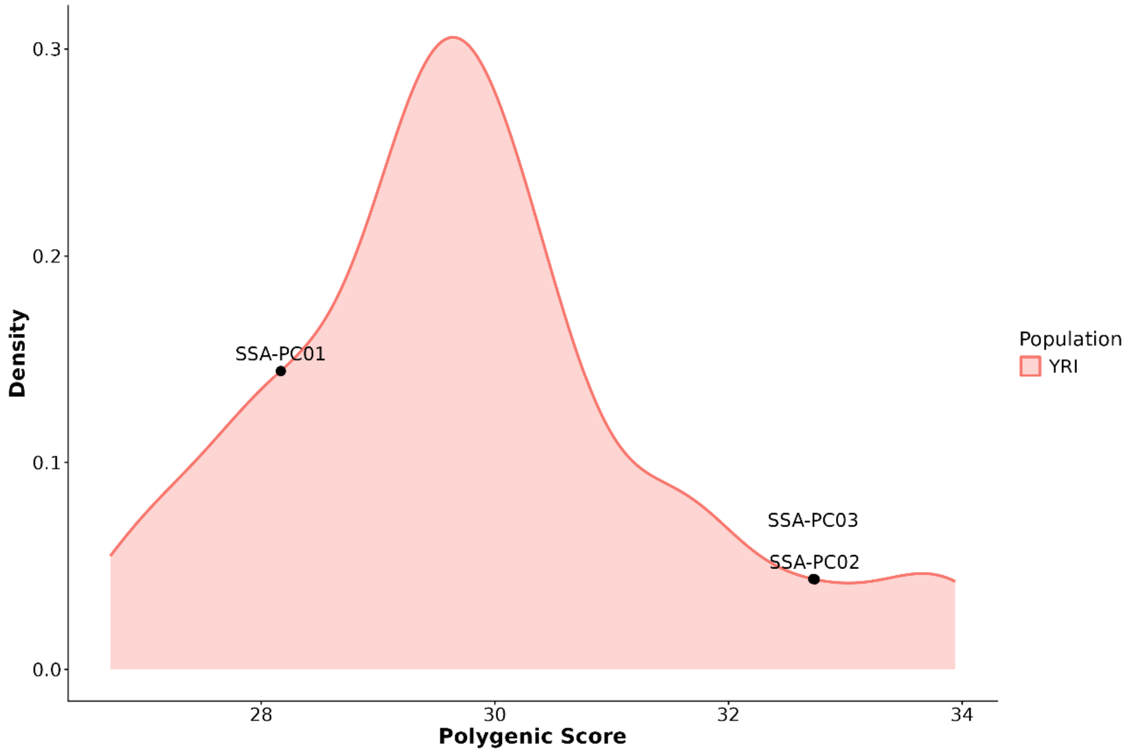

Supplementary Figure S2. Density curve for the polygenic risk scores (PRSs) based on Janivara et al. [23] for the Yoruba (YRI) male population from the 1kGP database and for the three patients from whom the SSA-PC cell lines were established.

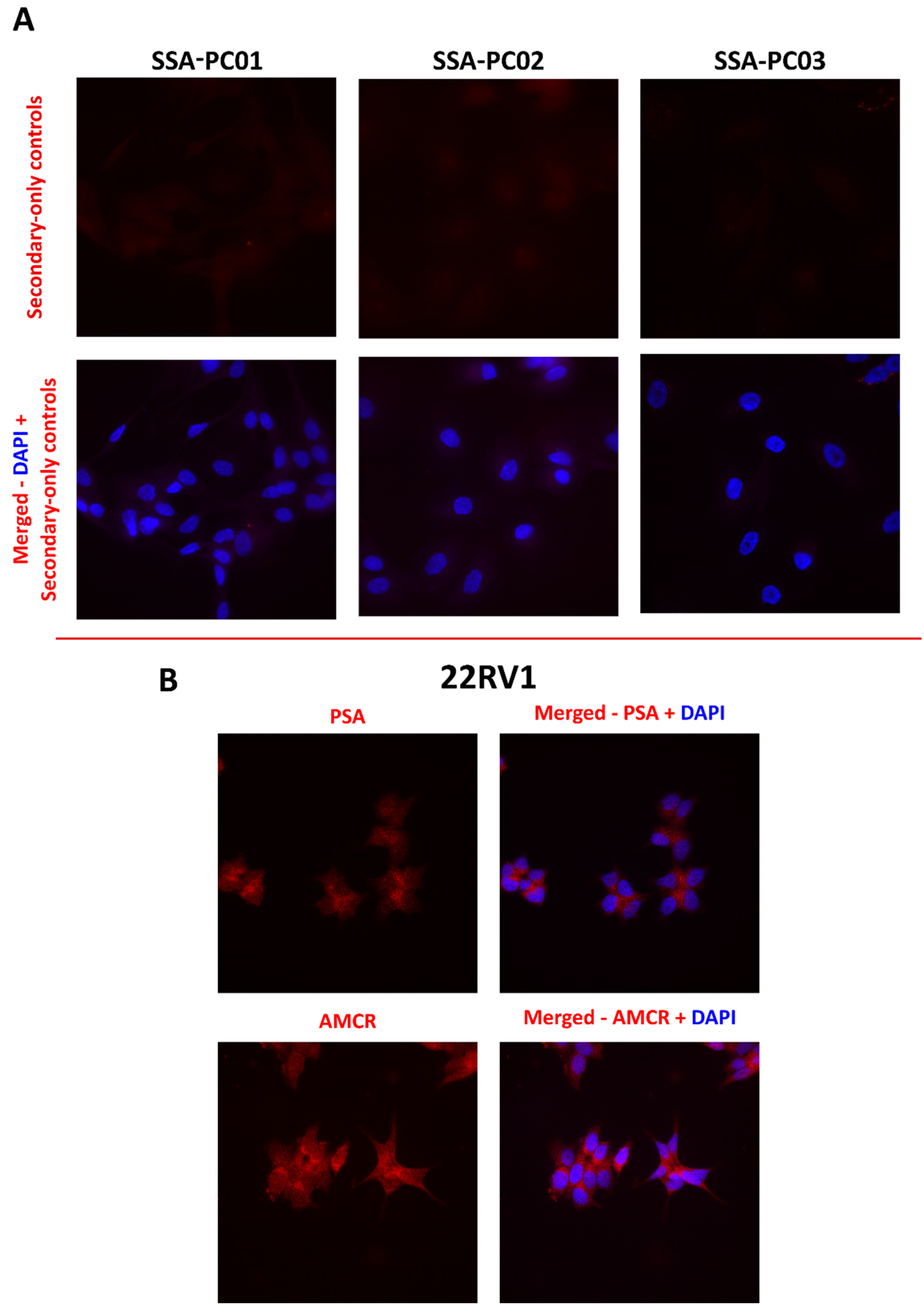

Supplementary Figure S3. Immunofluorescence controls for cytoplasmatic AMACR and PSA markers (both in red). (A) Negative controls for the SSA-PC cancer cell lines (secondary-antibody-only controls); (B) Positive controls for PSA and AMCR using the cell line 22RV1. All visualized with the inverted Leica DMI6000 microscope. Nuclei were stained with DAPI (blue).

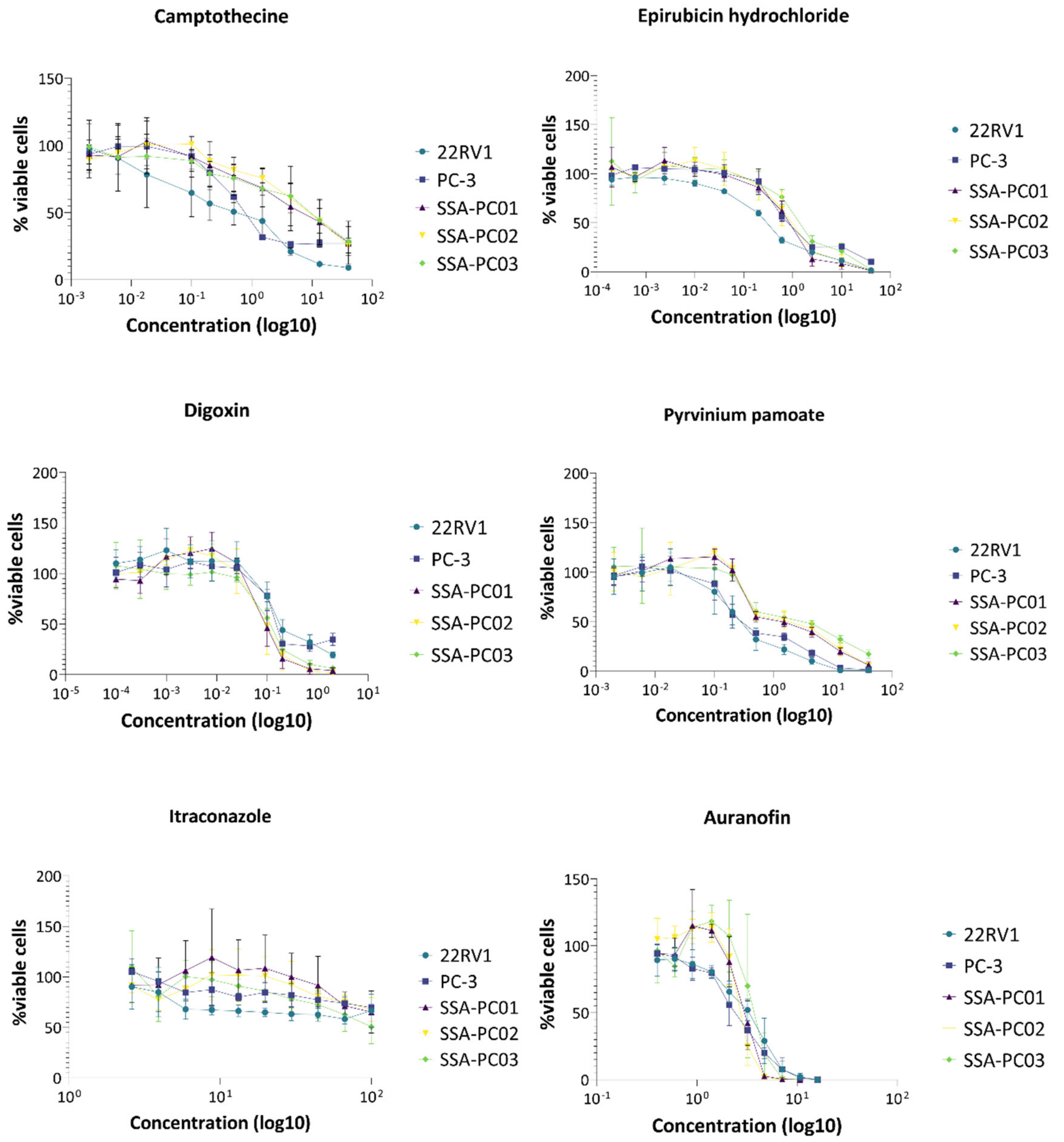

Supplementary Figure S4. IC50 calculation for six drugs in five cell lines. Cell viability across the ten different concentrations for the six drugs tested. Each cell line is represented with a different symbol and colour. Each point represents the mean of the three replicates. All standard deviations are also represented.
